# Case report: Decreased hemoglobin and multiple organ failure caused by ceftizoxime-induced immune hemolytic anemia in a Chinese patient with malignant rectal cancer

**DOI:** 10.3389/fimmu.2024.1390082

**Published:** 2024-05-02

**Authors:** Can Lou, Meng Liu, Ting Ma, Liu Yang, Dan Long, Jiaming Li, Hang Lei, Dong Xiang, Xuefeng Wang, Lei Li, Xiaohong Cai

**Affiliations:** ^1^ Department of Laboratory Medicine, Ruijin Hospital, Shanghai Jiao Tong University School of Medicine, Shanghai, China; ^2^ Department of Blood Transfusion, Ruijin Hospital, Shanghai Jiao Tong University School of Medicine, Shanghai, China; ^3^ Department of Critical Care Medicine, Ruijin Hospital, Shanghai Jiao Tong University School of Medicine, Shanghai, China; ^4^ Blood Group Reference Laboratory, Shanghai Institute of Blood Transfusion, Shanghai Blood Center, Shanghai, China; ^5^ Department of Blood Transfusion, Shaanxi Provincial People’s Hospital, Xian, Shaanxi, China; ^6^ Department of Blood Transfusion, The Second Affiliated Hospital of Guangxi Medical University, Nanning, China; ^7^ Department of Blood Transfusion, The Second Affiliated Hospital of Guizhou Medical University, Kaili, Guizhou, China

**Keywords:** DIIHA, ceftizoxime, direct antiglobulin test, antibody, decreased hemoglobin

## Abstract

**Background:**

Drug-induced immune hemolytic anemia (DIIHA) is a rare but serious condition, with an estimated incidence of one in 100,000 cases, associated with various antibiotics. This study reports on a case of ceftizoxime-induced hemolysis observed in a patient in China.

**Case description:**

A Chinese patient diagnosed with malignant rectal cancer underwent antimicrobial therapy after laparoscopic partial recto-sigmoid resection (L-Dixon). After receiving four doses of ceftizoxime, the patient developed symptoms including rash, itchy skin, and chest distress, followed by a rapid decline in hemoglobin levels, the presence of hemoglobin in the urine (hemoglobinuria), renal failure, and disseminated intravascular coagulation. Laboratory analysis revealed high-titer antibodies against ceftizoxime and red blood cells (RBCs) in the patient’s serum, including immunoglobulin M (IgM) (1:128) antibodies and immunoglobulin G (IgG) (1:8) antibodies, with noted crossreactivity to ceftriaxone. Significant improvement in the patient’s hemolytic symptoms was observed following immediate discontinuation of the drug, two plasma exchanges, and extensive RBC transfusion.

**Conclusion:**

This case, together with previous reports, underscores the importance of considering DIIHA in patients who exhibit unexplained decreases in hemoglobin levels following antibiotic therapy. A thorough examination of the patient’s medical history can provide crucial insights for diagnosing DIIHA. The effective management of DIIHA includes immediate cessation of the implicated drug, plasma exchange, and transfusion support based on the identification of specific drug-dependent antibodies through serological testing.

## Background

Drug-induced immune hemolytic anemia (DIIHA) is an adverse effect of some therapeutics with an incidence of 1 in 100,000, which can result in severe health complications, including drastically lowered hemoglobin levels, marked hemoglobinuria, renal failure, and even death (1, 2). The development of DIIHA is primarily driven by three mechanisms. The first is through drug-dependent antibodies. These are antibodies that target red blood cells (RBCs) in the presence of the implemented drug. There are two subtypes of drug-dependent mechanisms (3); (a) antibodies that react to drug-coated RBCs (i.e., penicillin-type DIIHA) (4) and (b) antibodies that form immune complexes in the presence of soluble drugs and subsequently bind to and damage RBCs, such as those seen with quinine or ceftriaxone (5). The second mechanism is through drug-independent autoantibodies. Unlike the first mechanism, this involves autoantibodies that attack RBCs regardless of the presence of a drug in the serum. Examples include autoantibodies associated with methyldopa, levodopa, and fludarabine (6, 7). Finally, the third mechanism is called nonimmune protein adsorption (NIPA). This mechanism is distinct from the previous two, as it occurs without direct antibody involvement and has been seen with cefotetan and oxaliplatin (8). In NIPA, the drug modifies the RBC membrane to allow non-specific adsorption of proteins to the cell surface, potentially leading to hemolysis without the traditional immune response (1, 3).

Historically, drugs like methyldopa and penicillin were frequently reported to induce DIIHA from the 1970s through the 1990s (6, 9). More recent literature has also identified cases linked to antibodies reacting to erythrocytes induced by newer drugs such as piperacillin, ceftibuten, and ceftriaxone (4–6). To date, around 140 different drugs have been identified as potential DIIHA triggers (3).

Detection methods for DIIHA vary based on the antibody type. For drug-dependent cases, such as those related to penicillin, aggregation tests using patient serum, the drug, and RBCs are effective (4, 9). Conversely, drug-independent autoantibodies, exemplified by those associated with methyldopa, react with untreated RBCs without the drug’s presence (6), making them challenging to differentiate from warm-type autoantibodies.

Only a limited number of drugs, including fludarabine, have been implicated in RBC aggregation via NIPA (7). Distinguishing between DIIHA caused by NIPA and drug-independent antibodies typically relies on the timing of hemolysis relative to drug administration (3, 7).

Cephalosporins, including widely prescribed antibiotics like ceftriaxone, and less commonly used agents such as ceftizoxime, have been associated with DIIHA due to drug-dependent antibodies (5), either through drug-coated cells or the formation of immune complexes (5, 10–15). The patients reported with ceftizoxime-induced DIIHA have had diverse underlying conditions, including rheumatic heart disease, diabetes mellitus, and metastatic cholangiocarcinoma (10–14, 16). This paper presents the first documented case of DIIHA triggered by ceftizoxime in a patient with malignant rectal cancer, exploring the specific mechanisms behind ceftizoxime-induced immune hemolytic anemia. The studies involving human participants were reviewed and approved by the ethics committee of Ruijin Hospital, affiliated with Shanghai Jiao Tong University School of Medicine. Written informed consent to participate in this study was provided by the patient.

## Case description

A 59-year-old Chinese Han woman, previously treated for a rectal polyp through polypectomy 2 years earlier, was later diagnosed with adenocarcinoma at the rectosigmoid junction. She was admitted for advanced care. On her second hospital day, she underwent laparoscopic partial resection of the recto-sigmoid colon (L-Dixon). During surgery, a tumor was found in the rectum, situated about 10 cm from the anal opening, exhibiting local invasion into the serosa and extending to the vaginal wall. The surgical team successfully performed a complete tumor excision with an end-to-end anastomosis connecting the distal colon and rectum. Pathological analysis postsurgery staged the tumor as pT4N2M0 and confirmed clear margins, indicating a complete (R0) resection.

The surgery proceeded smoothly without the need for a blood transfusion. However, a week postoperation, the patient developed symptoms of an anastomotic fistula, including abdominal pain. To manage and prevent potential infection, ceftizoxime (2 g every 12 h) was administered intravenously on days 9, 27, and 34 postadmission, leading to symptom improvement. On day 41, the patient experienced a recurrence of abdominal pain, prompting a fourth ceftizoxime dose. Roughly 15 min after this dose, she exhibited a rash, itching, and difficulty breathing, leading to the immediate cessation of the ceftizoxime infusion. Laboratory tests revealed an increased white blood cell count (WBC, 27.42 × 10^9^/L, with 87.3% neutrophils), reduced hemoglobin (Hb, 61 g/L), and a normal platelet count (PLT, 138 × 10^9^/L) (**Supplementary Table S1**). Given the drop in hemoglobin and potential bleeding concerns, an urgent exploratory laparotomy was performed on day 42, uncovering moderate dark red bloody ascites in the abdominal cavity with no evident pus or fecal-like material.

Coagulation tests indicated a disturbed clotting function, with both activated partial thromboplastin time (APTT, 72.3 s; normal reference, 22.3–38.7 s) and prothrombin time (PT, 46.0 s; normal reference, 10.0–16.0 s) showing prolongation. Fibrinogen (Fg) levels were low at 0.5 g/L, and both fibrin degradation products (FDP, 120 mg/L; normal reference, 0–5 mg/L) and d-dimer levels (40 mg/L; normal reference, 0–0.55 mg/L) were elevated, suggestive of significant coagulation dysfunction.

The patient’s transfusion history and reactions prior to hospital admission remained unknown, and blood typing identified her as A/Rh positive (CcEe). Throughout her hospital stay, all antibody screening tests were consistently negative. A treatment involving the transfusion of 12 units of RBCs and 1,400 ml of plasma was administered. Each unit of RBCs, with additives, approximates 150 ml, derived from 200 ml of whole blood, while a plasma unit in China measures 100 ml. This intervention led to a noticeable reduction in hemorrhagic exudate and enhanced the outcomes of coagulation function tests, demonstrating values of APTT at 55.8 s, PT at 18.4 s, Fg at 1.8 g/L, FDP at 82.1 mg/L, and d-dimer at 40 mg/L.

**Figure 1:**
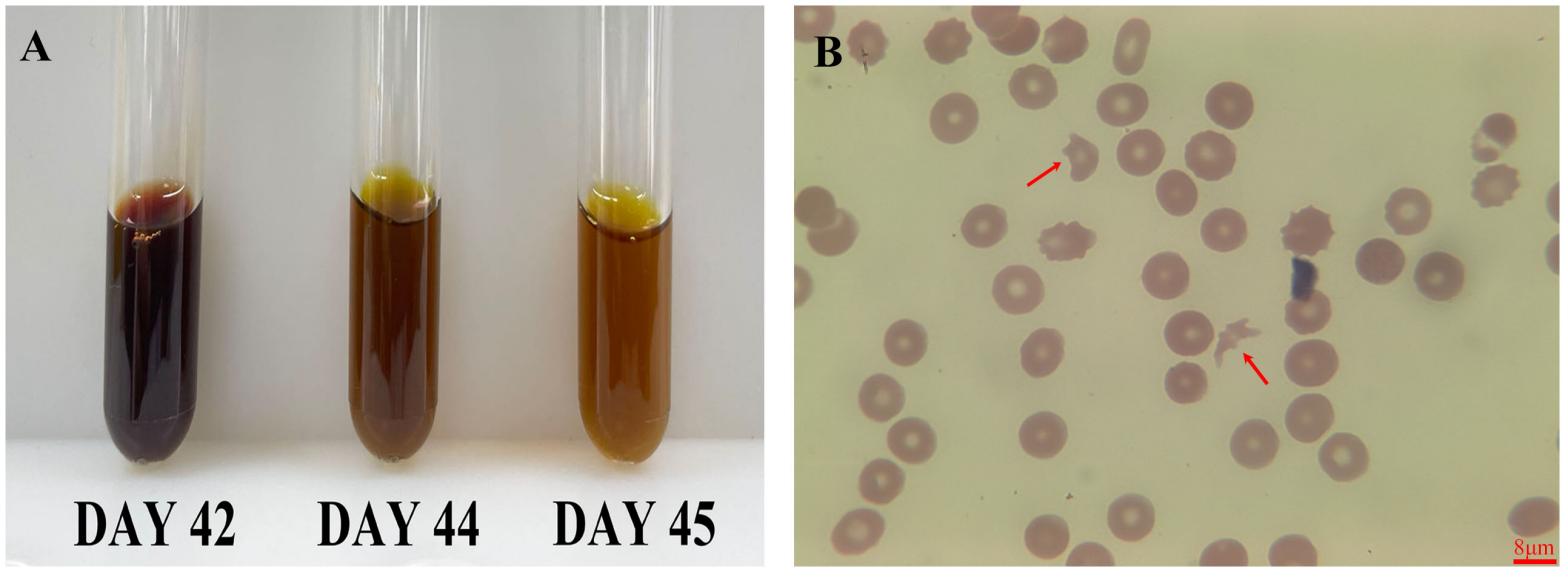
Serum and peripheral smear changes post-ceftizoxime. This figure showed the patient’s recovery from dark, soy sauce-colored serum to normal following ceftizoxime cessation **(A)**, and a peripheral blood smear revealed numerous schistocytes, indicating hemolysis (**B**, red arrow). The scale bar is 8 μm.

On the same day (day 42), the patient’s hemoglobin levels sharply declined to 53 g/L, accompanied by dark, soy sauce-colored urine. Concurrently, there was a significant surge in bilirubin—especially unconjugated bilirubin—alanine aminotransferase (ALT), aspartate aminotransferase (AST), and lactate dehydrogenase (LDH) levels. The patient’s plasma (or serum) mirrored the dark hue of soy sauce, and peripheral blood smears revealed an abundance of schistocytes, indicating active hemolysis (**Figure 1**). An abdominal CT scan detected fluid accumulation around the surgical site. Neither next-generation sequencing (NGS) for pathogenic organisms nor blood cultures taken on the 42nd day yielded positive results, thus ruling out infection.

**Figure 2:**
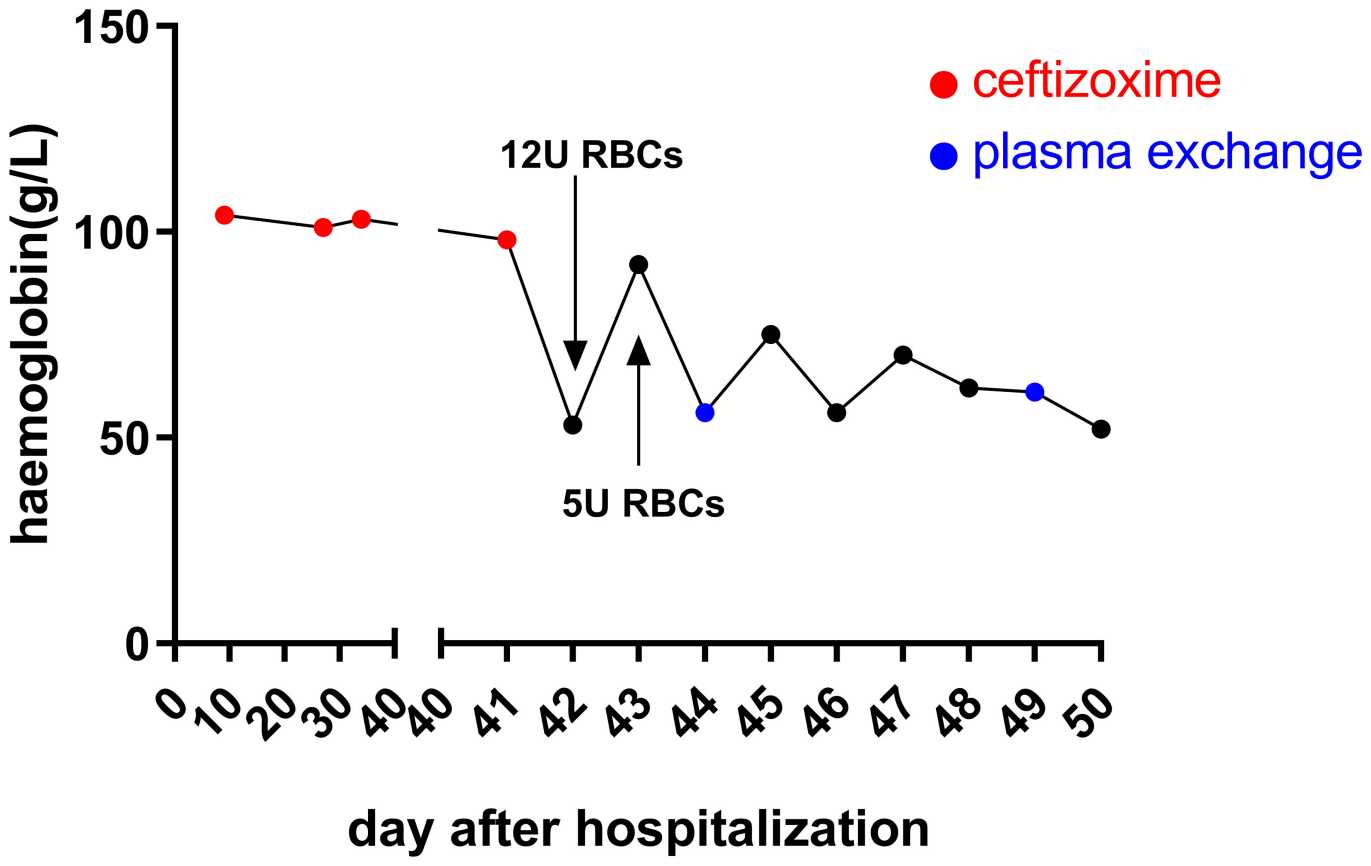
Hemoglobin levels after hospital admission. Upon admission, the patient’s hemoglobin levels remained consistent. However, following the last administration of ceftizoxime via an IV drip, a rapid decrease in hemoglobin was observed within 24 h. Although there was a temporary improvement in hemoglobin levels after receiving multiple RBC transfusions, the ongoing destruction of hemoglobin by drug-dependent antibodies led to a continued decrease. On days 44, 45, 46, and 49, the patient received 2 units of RBCs each day to combat this decline.

Posttransfusion, the patient’s hemoglobin improved to 92 g/L after receiving an additional 12 units of RBCs. Despite this, hemolysis continued, causing hemoglobin levels to fluctuate in the subsequent days (**Figure 2**). The patient reported chest pain following the transfusion. Markers of myocardial enzyme activity, such as creatine kinase isoenzymes (CK-MB) and cardiac troponin I (cTnI), escalated to 7.3 ng/ml (normal reference, 0.3–4 ng/ml) and 3,670.7 pg/ml (normal reference, 0–70 pg/ml), respectively, although the electrocardiogram only indicated sinus tachycardia without signs of myocardial infarction. Given the rapid onset of symptoms and decline in hemoglobin levels following ceftizoxime administration, ceftizoxime-induced DIIHA was strongly suspected. A battery of laboratory tests was subsequently performed to confirm the DIIHA diagnosis in the patient.

We evaluated blood samples using the direct antiglobulin test (DAT) (17) and found positive results in patient samples treated with EDTA anticoagulants. However, when we applied the indirect antiglobulin test (IAT) using the ORTHO VISION Analyzer (Ortho, Raritan, NJ, USA), the outcome was negative. Further analysis with a different classification system by Immucor (Norcross, GA, USA) identified the presence of C3 complement in the DAT but did not detect Immunoglobulin G antibody (IgG). Following this, we assessed the presence of drug-dependent antibodies in the serum of the patient exposed to ceftizoxime. This multistep testing process allowed us to closely examine the immune response related to ceftizoxime in the context of hemolytic reactions.

To assess the interaction between ceftizoxime and RBCs under conditions that mimic therapeutic drug levels, we employed a method based on guidelines by the Association for the Advancement of Blood and Biotherapies (AABB) (18). Initially, we prepared a 450-μl suspension of O-type RBCs and mixed it with 50 μl of a ceftizoxime solution (0.5 g of the drug dissolved in 50 ml of normal saline), achieving a final concentration of 1 mg/ml. This mixture was incubated at 37°C for 30 min and subsequently washed three times with saline to ensure the removal of unbound drug molecules. Following this, 100 μl of the patient’s serum was combined with 50 μl of the treated RBC suspension, and the mixture was immediately centrifuged (**Table 1**). This step did not result in RBC agglutination. However, adding 20 μl of soluble ceftizoxime to a mixture containing 120 μl of the patient’s serum and 60 μl of O-type RBCs led to visible agglutination after centrifugation.

**Table 1:** Agglutination reactions involving patient serum, drugs, and O-type RBCs.

Groups	Immediate spin (IS)	37°C	IgG (ortho anti-IgG, –C3d; polyspecific gel card)
Patient serum + ceftizoxime + O-type RBCs	4+	4+	Not applicable (N/A)
Patient serum + ceftizoxime-treated O-type RBCs	No agglutination (θ)	θ	θ
2-ME-treated patient serum + ceftizoxime + O-type RBCs	θ	θ	3+
2-ME-treated patient serum + ceftizoxime-treated O-type RBCs	θ	θ	θ
Patient serum + ceftriaxone + O-type RBCs	θ	4+	N/A
2-ME-treated patient serum + ceftriaxone + O-type RBCs	θ	θ	1+
Negative control 1: patient serum + O-type RBCs	θ	θ	θ
Negative control 2: AB-type donor serum + O-type RBCs + ceftizoxime/ceftriaxone	θ	θ	θ

Concentration of ceftizoxime/ceftriaxone: 1 mg/ml. Incubation: serum and 2-ME/ceftizoxime-treated RBCs were incubated at 37°C for 30 min. Agglutination intensity was graded from no agglutination (θ) to strong agglutination (4+).

Ps, patient’s serum; Oc, O-type RBCs; ABs, AB-type blood donor’s serum (without antibodies).

To verify our findings, we conducted two control experiments: one combined the patient’s serum with O-type RBCs, and the other used AB-type blood donor serum (lacking antibodies) with O-type RBCs and ceftizoxime. In both controls, no hemolysis occurred, even after the addition of fresh complement or using fresh serum from the patient.

**Figure 3:**
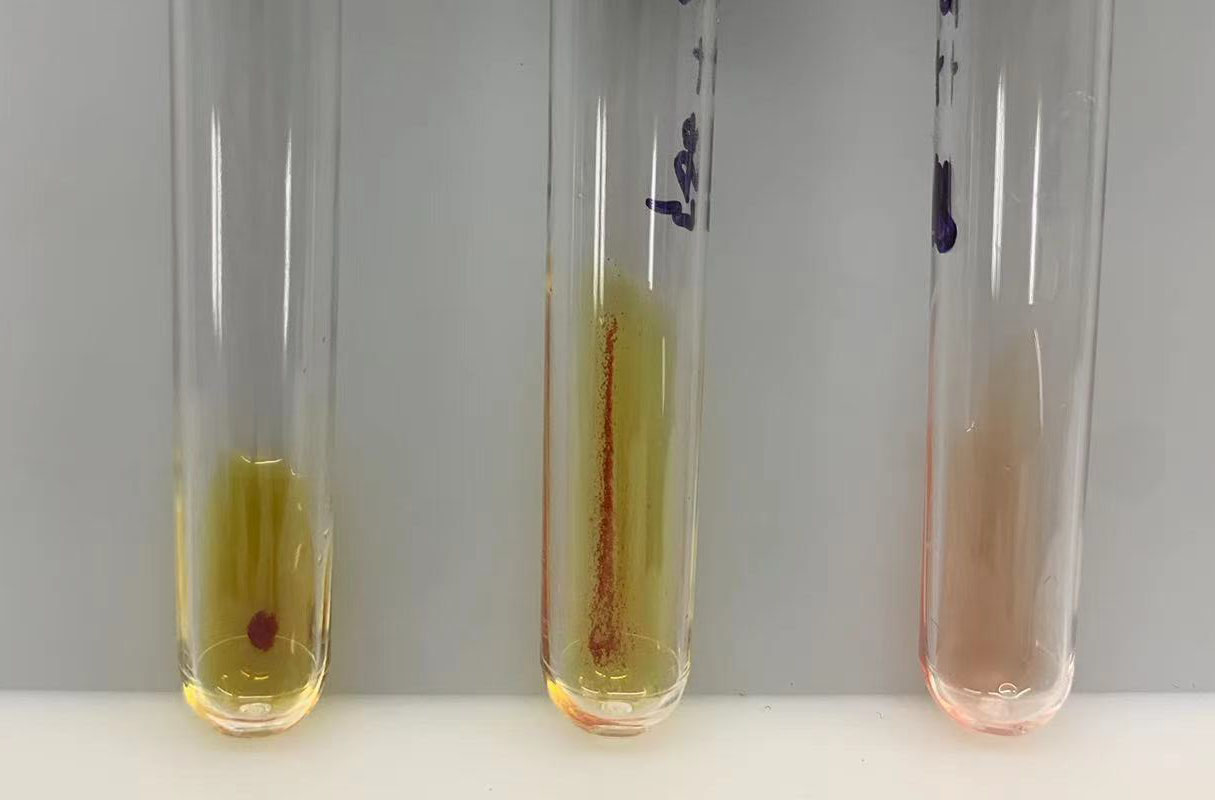
Agglutination responses to varying ceftizoxime concentrations. The agglutination responses elicited by different concentrations of ceftizoxime were demonstrated using uniform volumes of erythrocytes and the patient’s serum. Specifically, the ceftizoxime concentrations in the left and middle tubes were 8 mg/ml and 0.5 mg/ml, respectively, while the right tube contained AB serum without the drug. Our observations revealed a significantly stronger agglutination reaction in the tube with 8 mg/ml ceftizoxime compared to the one with 0.5 mg/ml, highlighting the dose-dependent nature of the agglutination process.

Further testing with varying concentrations of ceftizoxime revealed a direct correlation between the drug concentration and the intensity of agglutination (**Figure 3**). This increase in agglutination intensity with higher ceftizoxime concentrations suggested that the antibody interaction was drug concentration-dependent.

Based on these observations, we concluded that the antibodies induced by ceftizoxime can cause RBC agglutination in the presence of soluble drugs, indicating that the mechanism behind ceftizoxime-dependent DIIHA is mediated by immune complexes.

2-Mercaptoethanol (2-ME) is a sulfhydryl agent effective in dispersing immunoglobulin M (IgM) antibodies (18). Unlike IgM, IgG antibodies are not inactivated by 2-ME and retain their serological properties, including the ability to sensitize corresponding RBCs. While dispersed IgM antibody molecules can still bind to their antigen after being split into subunits, they lose the ability to cause agglutination with corresponding RBCs.

According to AABB methods (18), treatment of patient serum with 2-mercaptoethanol (Baso, Zhuhai, China) at 37°C for 30 min maintained the agglutination reaction when mixed with ceftizoxime and type O erythrocytes using an Ortho gel card (anti-IgG, anti-C3d; polyspecific) (Ortho, NJ, USA). This suggested the presence of IgG-type-induced antibodies as well. It was noted that relatively low-titer IgG antibodies coexist with higher-titer IgM antibodies. While it is understood that during an immune response, there can be a transition from IgM antibodies to IgG antibodies (19), no evidence supporting this transition was found in the present case. Upon gradient dilution of the patient’s serum with normal saline, antibody titers were determined as 1:128 for IgM antibodies and 1:8 for IgG antibodies using immediate centrifugation and Ortho gel card (anti-IgG, anti-C3d; polyspecific), respectively (**Table 2**). The titer determination was carried out as AABB’s serial dilution method (18).

**Table 2:** Ceftizoxime-induced IgM and IgG antibody titers posthospitalization.

Day	IgM titer (immediate spin)^a^	IgG titer (2-ME treatment)^a^
42	32	Not detected
44	32	Not detected
45	64	8
46	128	8

a The IgM antibody titers were determined using immediate spin techniques. For IgG antibodies, titers were identified after treating the patient’s serum with 2-mercaptoethanol (2-ME) for 30 min at 37°C, facilitating the identification of soluble antibodies. The ceftizoxime concentration in the patient’s serum was measured at 1 mg/ml. “Not detected” indicates no agglutination reaction was observed.

To investigate whether the induced antibody could bind to platelets, causing immune-mediated destruction of platelets, a mix of type O platelets, capture-P (Immucor, Norcross, GA, USA), and patient serum were analyzed using a Galileo Neo instrument (Immucor, Norcross, GA, USA). The ceftizoxime-induced antibodies were found not to bind to platelets.

In the realm of clinical therapy, while hundreds of drugs are administered via infusion, only a few recipients develop detectable antibodies against these drugs. This immune response hinges on exposure to the drug and the presence of specific human leukocyte antigen (HLA) II types that can present parts of the drug–RBC complexes as foreign. Some HLA class II genotypes have been reported to be associated with the production of autoantibodies and alloantibodies (20–22). Notably, our investigation into the HLA types of this patient revealed DRB1*04:05, which was significantly more common among patients with autoimmune hepatitis (AIH), alongside other alleles such as *DRB4* and DQB1*04:01. This suggests that some people may share some specific *HLA-DRB* alleles, which could potentially influence how their immune system reacts to drug–RBC immune complexes. To verify this hypothesis, further investigation involving more cases is required.

Remarkably, the ceftizoxime-induced antibodies demonstrated crossreactivity with ceftriaxone at 37°C, causing agglutination of RBCs in the presence of soluble ceftriaxone (2 g, China). No agglutination was observed during immediate centrifugation at room temperature, and only weak agglutination intensity was noted in 2-ME-treated serum in the presence of soluble ceftriaxone. On day 48, the patient’s DAT turned negative, and the hemolytic symptoms significantly improved after the prompt discontinuation of the drug, accompanied by two plasma replacements and extensive RBC transfusions.

## Conclusion

Immune hemolytic anemia arises from the destruction of RBCs by specific antibodies, leading to symptoms such as chest discomfort, difficulty breathing, renal failure, and, in severe cases, death. While rare, DIIHA is primarily associated with three immune mechanisms: drug-dependent antibodies, drug-independent antibodies, and nonimmune mechanisms, such as NIPA. Diagnosis of DIIHA includes confirmation of the temporal relationship between the drug and hemolysis, a possibly positive DAT, and the potential drug-induced antibodies.

Referencing Wu (16), our study presents a case of ceftizoxime-induced immune hemolytic anemia in a Chinese patient, further supporting DIIHA’s laboratory-based diagnosis. Ceftizoxime, akin to ceftriaxone, is a third-generation cephalosporin previously identified to trigger immune hemolytic anemia through drug-dependent antibodies, as highlighted in earlier research (10, 12, 14). Similar to previously reported cases (10, 12, 14), our study encountered severe hemolysis driven by immune complexes. This underscores the necessity to suspect DIIHA with any unexpected decrease in hemoglobin levels during antibiotic treatment. We recommend promptly halting the implicated antibiotic upon serologically identifying specific drug-dependent antibodies, followed by therapeutic plasma exchange and supportive transfusion as in our case. This intervention, timely executed, did not adversely affect the compatibility of further transfusions, culminating in effective DIIHA management. A comparative analysis of our patients against those in previous reports is detailed in **Table 3**, illustrating consistent findings across cases.

**Table 3:** Comparative analysis of ceftizoxime-induced immune hemolytic anemia cases.

Case reference	Clinical diagnosis	Gender	Ceftizoxime dosage	Duration of administration (days)	IgM titer	IgG titer	Times of plasma exchange	Survival days after discontinuation	Total units of component transfusion
Current case	Rectal cancer	Woman	2 g/day	4	128	8	2	66	56U RBCs + 9 doses AP + 5,300 ml plasma
Calhoun et al. (2001) (12)	Chronic bronchitis	Man	2–6 g/day	3	64	32	0	1	9U RBCs
Endoh et al. (1999) (10)	Cholangiocarcinoma	Man	1 g/day	23	Not available (N/A)	N/A	4	14	N/A

AP, apheresis platelets.

In this case, the patient exhibited severe symptoms and developed an immune reaction after repeated exposure to ceftizoxime, leading to the generation of specific antibodies targeting RBCs. This immune response triggered acute intravascular hemolysis, which was characterized by the fragmentation of RBCs, increased reticulocyte count, reduced haptoglobin levels, and the presence of hemoglobin in the urine. As a result, free heme and large fragments of erythrocytes were released through the kidneys, significantly compromising renal function and culminating in renal failure.

Throughout the disease progression in our case, there was an observed increase in IgM antibodies, followed by the emergence of IgG antibodies. High levels of IgM antibodies and lower levels of IgG antibodies were detected in the patient’s serum in the presence of O-type RBCs and ceftizoxime. Given the structural similarities among cephalosporins and their mechanisms causing DIIHA, we assessed the agglutination reaction with third-generation cephalosporins, including ceftizoxime, ceftriaxone, and ceftazidine, as well as the first-generation cephalosporin, cefazolin. We found that the ceftizoxime-induced antibodies exhibited only mild cross-reactivity with ceftriaxone. Consistent with prior research (1), antibodies induced by ceftriaxone showed cross-reactivity with ceftizoxime but not with cefazolin or ceftazidime. Nonetheless, it is advised to discontinue all cephalosporins upon diagnosing cephalosporin-induced hemolysis.

To further confirm the specificity of the antibody to human RBCs, we also conducted tests using human platelets, chicken RBCs, and sheep RBCs. The findings revealed that the ceftizoxime-dependent antibodies did not bind to platelets, chicken RBCs, or sheep RBCs (data not shown), indicating that the ceftizoxime-induced antibodies target universal structures on human RBCs in the presence of soluble ceftizoxime.

The patient’s hemolytic anemia was effectively managed following the immediate discontinuation of ceftizoxime, complemented by two plasma exchanges and RBC transfusion. Plasma exchanges were performed on days 44 and 49, with 2,000 ml of plasma exchanged via apheresis each time. The schedule for RBC transfusion, as well as plasma exchange details, are provided in **Supplementary Table S2**. Although the pathogenic investigations were negative on the 42nd day of hospitalization, from day 45 onwards, *Enterococcus faecalis*, *Morgenella morgensis*, *Candida alba*, *Staphylococcus aureus*, and *Acinetobacter baumannii* were successively identified in the patient’s drainage fluid through NGS, or blood culture. Approximately 100 days postadmission, head CT scans (**Supplementary Figure S1**), myocardial markers (such as CK-MB and cTnI, escalated to 282.4 ng/ml and 22,231.3 pg/ml), and electrocardiogram results indicated the development of a significant cerebral infarction and myocardial infarction. Unfortunately, on day 107, the patient succumbed to complications of the underlying disease.

This case, along with others, emphasizes the need to consider DIIHA in patients presenting with unexplained reductions in hemoglobin levels and a history of antibiotic use. The detection of specific drug-dependent antibodies through serologic tests necessitates immediate drug discontinuation, plasma exchange, and supportive transfusion-related interventions.

## Data availability statement

The original contributions presented in the study are included in the article/**Supplementary Material**. Further inquiries can be directed to the corresponding authors.

## Ethics statement

The studies involving humans were approved by The Ruijin Hospital Ethics Committee. The studies were conducted in accordance with the local legislation and institutional requirements. The participants provided their written informed consent to participate in this study. Written informed consent was obtained from the participant/patient(s) for the publication of this case report.

## Author contributions

CL: Writing – original draft. ML: Writing – original draft. TM: Writing – original draft. LY: Writing – original draft. DL: Writing – original draft. JL: Writing – review & editing. HL: Writing – review & editing. DX: Writing – review & editing. XW: Writing – review & editing. LL: Writing – review & editing. XC: Writing – review & editing.
