# Supplementary material for: Case report: Decreased hemoglobin and multiple organ failure caused by ceftizoxime-induced immune hemolytic anemia in a Chinese patient with malignant rectal cancer
Source: Front Immunol. 2024 May 2;15:1390082. doi: 10.3389/fimmu.2024.1390082 (PMC11096485; doi:10.3389/fimmu.2024.1390082)
Supplement: Supplementary file 1 [file DataSheet_1.pdf]

Table S1: Laboratory Data for the Patient Before and After the 4th Dose of Ceftizoxime.

Parameter	Before (Units)	After (Units)	Reference Range (Units)
WBC (White Blood Cells)	6.33 (x10^9/L)	36.46 (x10^9/L)	3.5-9.5 (x10^9/L)
HGB (Hemoglobin)	108 (g/L)	53 (g/L)	115-150 (g/L)
HCT (Hematocrit)	0.36	0.14	0.35-0.45
PLT (Platelet Count)	308 (x10^9/L)	49 (x10^9/L)	125-350 (x10^9/L)
BUN (Blood Urea Nitrogen)	3.1 (mmol/L)	18.2 (mmol/L)	2.5-7.1 (mmol/L)
CR (Creatinine)	46 (μmol/L)	241 (μmol/L)	53-97 (μmol/L)
TB (Total Bilirubin)	17.2 (μmol/L)	270.5 (μmol/L)	4.7-24 (μmol/L)
DB (Direct Bilirubin)	3.8 (μmol/L)	117.4 (μmol/L)	0-6.8 (μmol/L)
HPT (Haptoglobin)	NA	0.03 (g/L)	0-11.4 (g/L)
LDH (Lactate Dehydrogenase)	169 (IU/L)	12620 (IU/L)	120-250 (IU/L)
PT (Prothrombin Time)	11.8 (s)	46 (s)	10-16 (s)
INR (International Normalized Ratio)	1.01	4.41	/
APTT (Activated Partial Thromboplastin Time)	31.2 (s)	72.3 (s)	22.3-38.7 (s)
FG (Fibrinogen)	2.5 (g/L)	BDL	1.8-3.5 (g/L)
DD (D-dimer)	1.54 (mg/L)	40 (mg/L)	0-0.55 (mg/L)
FDP (Fibrin Degradation Products)	5.2 (mg/L)	120 (mg/L)	0-5 (mg/L)
RBC in urine (Microscopic result)	11 (/μL)	2662 (/μL)	0-25 (/μL)
CK-MB (Creatine kinase isoenzymes)	0.3 (ng/ml)	7.3 (ng/ml)	0.3-4 (ng/ml)
cTnI (Cardiac troponin I)	1.8 (pg/ml)	3670.7 (pg/ml)	0-70 (pg/ml)

NA: Not Available; BDL: Below Detection Limit.

Table S2: Timeline of Blood Component Transfusions and Plasma Exchange in the Course of and Following DIIHA Episode in the Patient.

Day	Red Blood Cell (RBC) Units	Apheresis Platelets (AP) Doses	Plasma Exchange (PE)	Plasma
42	12 Units	1 Dose	-	1400 ml
43	5 Units	-	-	1200 ml
44	2 Units	2 Doses	1st (2000 ml)	-
45	2 Units	2 Doses	-	-
46	2 Units	-	-	200 ml
49	2 Units	1 Dose	2nd (2000 ml)	-
51	2 Units	1 Dose	-	200 ml
56	4 Units	-	-	-
Day 56 to Day 107 (Death)	25 Units	2 Doses	-	2300 ml

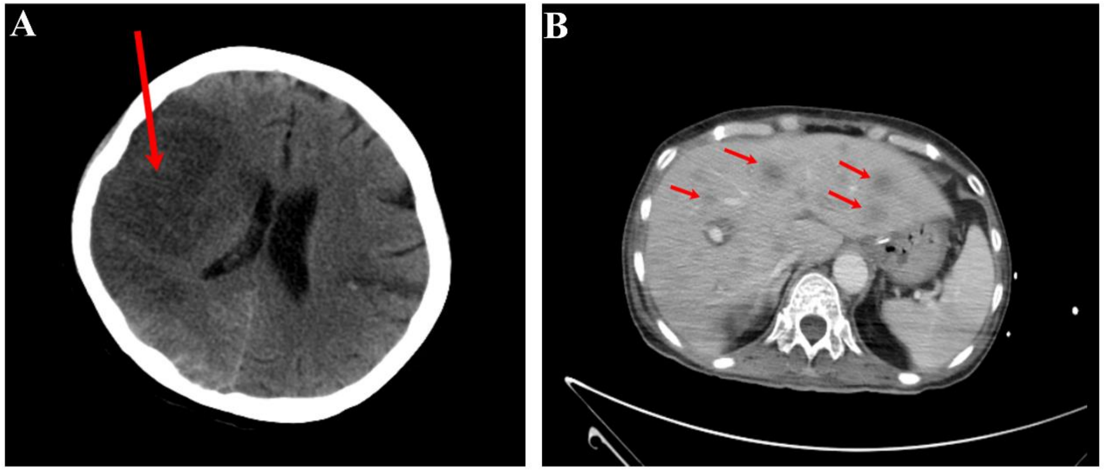

Figure S1.CT Scan results of Hepatic Metastases and Cerebral Infarction. (A) On the 104th day following hospital admission, a craniocerebral CT scan revealed a prominent low-density lesion on the right cerebral hemisphere, consistent with a cerebral infarction. (B) Concurrently, a detailed CT scan of the patient's abdomen was performed, identifying numerous diffuse hepatic metastases during the portal phase, indicated by a red arrow.
